# Exploration and Enrichment Analysis of the QTLome for Important Traits in Livestock Species

**DOI:** 10.3390/genes15121513

**Published:** 2024-11-26

**Authors:** Francisco J. Jahuey-Martínez, José A. Martínez-Quintana, Felipe A. Rodríguez-Almeida, Gaspar M. Parra-Bracamonte

**Affiliations:** 1 Facultad de Zootecnia y Ecología, Universidad Autónoma de Chihuahua, Chihuahua 31453, Mexico; jomartinez@uach.mx (J.A.M.-Q.); frodrigu@uach.mx (F.A.R.-A.); 2 Centro de Biotecnología Genómica, Instituto Politécnico Nacional, Reynosa 88710, Mexico; gparra@ipn.mx

**Keywords:** QTLome, quantitative trait loci, livestock species, enrichment analysis, productive traits

## Abstract

**Background:** Quantitative trait loci (QTL) are genomic regions that influence essential traits in livestock. Understanding QTL distribution and density across species’ genomes is crucial for animal genetics research. **Objectives:** This study explored the QTLome of cattle, pigs, sheep, and chickens by analyzing QTL distribution and evaluating the correlation between QTL, gene density, and chromosome size with the aim to identify QTL-enriched genomic regions. Methods: Data from 211,715 QTL (1994–2021) were retrieved from the AnimalQTLdb and analyzed using R software v4.2.1. Unique QTL annotations were identified, and redundant or inconsistent data were removed. Statistical analyses included Pearson correlations and binomial, hypergeometric, and bootstrap-based enrichment tests. **Results:** QTL densities per Mbp were 10 for bovine, 4 for pig, 1 for sheep, and 3 for chicken genomes. Analysis of QTL distribution across chromosomes revealed uneven patterns, with certain regions enriched for QTL. Correlation analysis revealed a strong positive relationship between QTL and gene density/chromosome size across all species (*p* < 0.05). Enrichment analysis identified pleiotropic regions, where QTL affect multiple traits, often aligning with known candidate and major genes. Significant QTL-enriched windows (*p* < 0.05) were detected, with 699 (187), 355 (68), 50 (15), and 38 (17) genomic windows for cattle, pigs, sheep, and chickens, respectively, associated with overall traits (and specific phenotypic categories). **Conclusions:** This study provides critical insights into QTL distribution and its correlation with gene density, offering valuable data for advancing genetic research in livestock species. The identification of QTL-enriched regions also highlights key areas for future exploration in trait improvement programs.

## 1. Introduction

Quantitative trait loci (QTL) are genomic regions that influence phenotypic variation. Identifying these regions has been a topic of great interest in both animal and plant genetics. Research has focused on searching for QTL associated with various important production traits, such as growth, health, quality, and reproduction. In recent years, advancements in genotyping and sequencing technologies have accelerated the identification of QTL, shifting from the traditional candidate gene approach to genome-wide association studies (GWAS) [1]. This transition has led to the identification of hundreds of QTL [1], unveiling the QTLome for productive traits in livestock species. The QTLome includes information about the position, effect, and gene action of each QTL [2].

Typically, findings from genetic mapping studies are deposited in databases such as AnimalQTLdb [3]. This database consolidates QTL data for major livestock species, including cattle, horses, pigs, sheep, goats, chickens, and rainbow trout. As of 2023, AnimalQTLdb version 51 contains a total of 278,738 QTL associated with 2135 phenotypic traits from 2725 scientific studies. Over the years, this database has undergone improvements in its structure, content, and utility [4,5,6,7], becoming a valuable repository of biological information [8,9]. It has also served as a reference for meta-analysis studies [10,11,12] and gene prioritization [13,14], making it an essential resource for genetic mapping studies [15].

Despite its significance and enrichment, the QTL database has been relatively underutilized when it comes to exploring the QTLome of quantitative traits. While some scientific reviews of QTL have focused on specific traits such as milk [8,16] and fertility [9] in cattle or fat deposition in pigs [17], more comprehensive studies have been conducted in other species, like plants [18] and fish [19], revealing that many phenotypic traits are polygenic and controlled by multiple loci across the genome. Notably, in cattle, certain chromosome segments exhibit pleiotropic effects on multiple traits [20,21,22], while others appear specialized for specific phenotypic traits [23,24]. These studies, combined with the prioritization and exploration of genomic regions containing QTL, have identified biologically relevant genes associated with productive traits [13,14,17,23,25].

Therefore, the objective of this research was to analyze the genomic distribution of QTL in four livestock species (cattle, pigs, sheep, and chickens) and identify key genomic regions associated with phenotypic traits through enrichment analysis. Our hypotheses regarding QTLome properties are as follows: (1) Since each gene could be a QTL, the distribution of QTL may be associated with the distribution of genes. Therefore, (2) regions with more genes may have a higher number of QTL. Finally, (3) QTL-enriched regions may be associated with a greater number of phenotypes.

## 2. Materials and Methods

In this study, we utilized QTL information from the AnimalQTLdb database version 44 [7], which comprises over 216,000 annotations for six animal species. Only QTL data for cattle, pigs, sheep, and chickens were utilized because these species had the highest number of QTL in the database, and their annotations were assigned to positions in the reference genomes: UMD 3.1, SS 10.2, GG 5.0, and OAR 3.1, respectively. Genome and short variant data for each species were obtained from Ensembl website release 94 (http://oct2018.archive.ensembl.org/index.html, accessed on 31 January 2022). QTL annotation files in general feature format (gff) were downloaded from AnimalQTLdb (https://www.animalgenome.org/cgi-bin/QTLdb/index, accessed on 26 April 2021).

Each database represented QTL as sets of genomic intervals with their respective chromosomal coordinates, along with additional metadata, including the original research identifier, the animal breed in which the QTL was discovered, and the associated phenotypic traits. We opted for version 44 of the AnimalQTLdb because it retained the original trait names from the original papers. In newer database updates, these traits had been merged into new categories, which would have interfered with the objectives of this study.

### 2.1. Quality Control of Databases

The gff files for the QTL of each species were imported into R software v4.2.1 and analyzed using a series of libraries, as described below. An exploratory analysis of each dataset was conducted using the GenomicRanges package [26] to assess annotation quality, examining QTL locations, their associations with phenotypic traits, and the number of QTL reported by each study. Annotations without coordinates in the reference genomes or with anomalous locations (e.g., start = 0 and end = 100) or locations outside chromosomal limits were initially excluded. QTL longer than 10 megabase pairs (Mbp) were also removed. From the remaining data, duplicated annotations were identified and removed, retaining only unique annotations. Additionally, similar annotations were identified and merged, that is, combining QTL that were entirely or partially positioned within other QTL by at least one base pair (bp) or separated by less than 500 kilo base pairs (kbp) but associated with the same phenotypic trait from the same study. Clustering of these annotations was conducted unless the new merged segments exceeded 1 Mbp in length. When the number or size of QTL was substantial, 1 Mbp windows with the highest QTL density were selected, covering at least 30% of the original total segment. These procedures significantly reduced the number of annotations. Finally, QTL occupying short genomic segments (e.g., single nucleotide polymorphisms or SNP) were expanded to 250 kbp, while those with excessive lengths were trimmed to 1 Mbp, achieving standardized QTL sizes within this defined range. All modifications were made while preserving the distribution patterns of QTL found in each database.

### 2.2. Analysis of the Distribution of QTL at the Genomic Level

To visualize the distribution of QTL and assess the current status of the QTLomes, we generated density graphs of annotations in each genome. Karyograms were created using the shinyCircos web tool [27], allowing comparison of QTL distribution with gene and SNP density, as well as the positions of major genes reported in the OMIA database version 2022 (https://omia.org/home/, accessed on 31 January 2022) [28]. This was done to detect potential association patterns, since previous QTLome studies in different plant [18] and animal species [19], including cattle [10], have reported relationships among these variables. QTL density was also visualized by chromosome and linear karyograms using the ggridges library [29] to improve observation of regions enriched with QTL.

In some QTLome studies [18,19], genome size has been identified as a good predictor of the number of QTL. Consequently, we investigated the correlation between the number of QTL and chromosome length ($r_{QTL,\;chr\;size}$) and the number of genes ($r_{QTL,\;genes}$) per chromosome to determine the extent to which these variables explained QTL distribution at the genomic level. Scatter plots were generated to visualize the relationship between these variables. To quantify the magnitude of this relationship at the chromosomal level, the number (density) of genes and QTL present in genomic windows of 0.25, 1, 2.5, and 5 Mbp was compared, and their correlation was assessed for each chromosome ($r_{0.25},\;r_{1},\;r_{2.5},\;r_{5}$). Chromosomes with fewer than 10 windows were excluded. Additionally, the average of chromosomal correlations ($\bar{r}$) was calculated, serving as a genomic indicator to evaluate the linear relationship between QTL density and gene density across different resolution scales, ranging from 0.25 to 5 Mbp (${\bar{r}}_{0.25},\;{\bar{r}}_{1},\;{\bar{r}}_{2.5},\;{\bar{r}}_{5}$). All correlation analyses employed the Pearson method, and *p*-values were obtained through permutation (*n* = 1000) with the permcor function of the MXM package [30]. Chromosomal correlations were plotted in a matrix using the corrplot package [31].

### 2.3. General QTL Enrichment Analysis

After determining that both gene number and chromosome size significantly accounted for the variation in the number of QTL at the genome level, hypothesis tests were conducted to identify which chromosomes were enriched or depleted for QTL, using chromosome size as the explanatory variable. This analysis involved comparing the observed number of QTL with the expected number using an exact binomial test [19]. The binomial distribution for each chromosome was determined by n (observed number of QTL) and p (proportion of the genome occupied by the chromosome, calculated as chromosome size/genome size). The expected number of QTL on each chromosome was then calculated as n × p, representing the product of the total QTL (n) and the relative size of the chromosome (p). A two-tailed hypothesis test was used with the binom.test function in R. This analysis was also performed at the regional level to identify genomic segments enriched with QTL, using the proportion of genes present in the window (number of genes in the window/total genes) as p. Regional enrichment analysis was conducted using QTL counts within 1 Mbp genomic windows.

To assess whether the increase in the number of QTL coincided with genomic regions containing major genes or commonly reported candidate genes, we conducted enrichment analyses by resampling QTL counts with the regioneR package [32]. We counted the overlaps between QTL and the genes of interest, comparing them with overlaps with genes or segments randomly obtained from the genome. The permTest function was used with 1000 permutations, and significance was considered at a *p*-value < 0.05. For this test, only unique QTL (non-repeated annotations) were used, and their size was set to 250 kbp, considering the midpoint of the QTL. The genes of interest were selected from the OMIA database version 2022 (https://omia.org/home/, accessed on 31 January 2022) [28] for each species, as well as the top 30 candidate genes with the highest number of reports in AnimalQTLdb version 44 [7] for each species. Tables S1 and S2 contain the complete list of genes used for this analysis. Among the major genes in the list are *ADAM* metallopeptidase with thrombospondin type 1 motif 2 (*ADMATS2*), bone morphogenetic protein 15 (*BMP15*), integrin subunit beta 4 (*ITGB4*), *KIT* proto-oncogene, receptor tyrosine kinase (*KIT*), melanocortin 1 receptor (*MC1R*), myostatin (*MSTN*), prolactin receptor (*PRLR*), prion protein (*PRNP*), glycogen phosphorylase-muscle associated (*PYGM*), tyrosinase (*TYR*), tyrosinase-related protein 1 (*TYRP1*), and others, which were present in at least two of the four species. Among the candidate genes used for enrichment analysis are acetyl-CoA carboxylase alpha (*ACACA*), calpastatin (*CAST*), diacylglycerol O-acyltransferase 1 (*DGAT1*), fatty acid synthase (*FASN*), growth hormone receptor (*GHR*), growth hormone secretagogue receptor (*GHSR*), leptin (*LEP*), leptin receptor (*LEPR*), prolactin receptor (*PRLR*), and stearoyl-CoA desaturase (*SCD*), also found in at least two of the four species.

### 2.4. QTL Enrichment Analysis for Each Phenotypic Trait

In another regional analysis, we utilized the hierarchical phenotypic categories from the AnimalQTLdb to identify genomic segments enriched with QTL associated with specific phenotypes (class, type, or trait). For each phenotypic category, we counted QTL within 1 Mbp windows and conducted a Fisher exact test. The phyper function was employed, where q represented the set of QTL for the class, type, or trait of interest within the genomic window—1, m was the total number of QTL in the window, n was the entire QTL database minus m, and k was the total number of QTL for the class, type, or trait of interest in the entire database. Genomic segments that showed significant enrichment in the regional analysis were displayed in Manhattan plots. In all enrichment analyses, *p*-values were adjusted using the Benjamini-Hochberg method through the *p* adjust function in R.

## 3. Results

A total of 211,715 QTL from the AnimalQTLdb database (version 44) were analyzed. These annotations are the result of research conducted from 1994 to 2021 across various animal breeds and populations, primarily stemming from candidate gene studies and GWAS. The length of the QTL ranged from two base pairs to 290.25 million base pairs (Mbp), with a median length of 1,020,303 base pairs (bp). However, the majority of the QTL (168,008) were associated with SNP types. Upon exploring the database, several observations were made: 114,621 QTL were identified as repeated annotations, meaning they were located in the same position, associated with the same characteristics, and originating from the same research; 15,149 QTL lacked chromosomal coordinates or had unusual positions; 4378 QTL had lengths exceeding 10 Mbp; and 1212 QTL were located or extended significantly beyond the chromosome boundaries. After applying rigorous quality control measures and grouping similar annotations, the number of QTL was substantially reduced to approximately 25% of the original count, resulting in a final count of 53,375 QTL. The total number of QTL per species before and after quality control is presented in Table 1. Overall, 76.4% (69.6%) of the QTL originated from cattle, 15.3% (20.3%) from pigs, 6.6% (5.8%) from chickens, and 1.7% (4.3%) from sheep.

### 3.1. Graphical Exploration of Livestock QTLome

**Figure 1:**
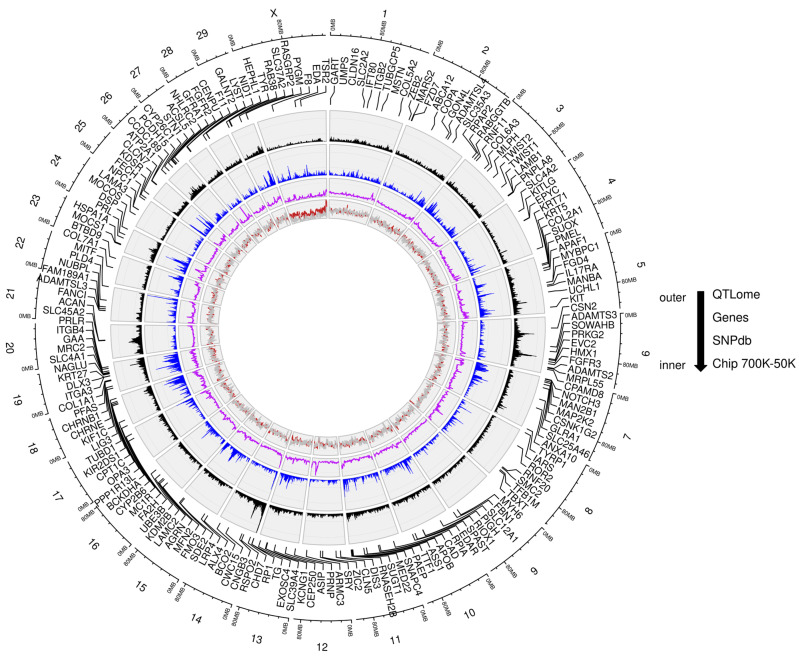
Circular karyogram of the bovine QTLome. The first layer depicts the locations of major genes (sourced from the OMIA database), while the subsequent layers illustrate the distribution of QTL (black line), genes (blue line), and SNP from the SNPdb (purple line) and genotyping microarrays commonly used in genetic mapping studies (gray and red lines). The density of QTL, genes, and SNP is represented as the number of annotations per Mbp. Chromosomes are indicated by numbers.

**Figure 2:**
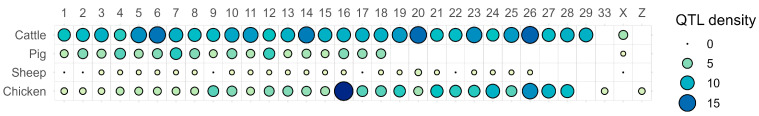
QTL density per chromosome in bovine, pig, sheep, and chicken genomes. The size and color of the circles represent the median number of QTL within 1 Mbp segments (QTL density) for each chromosome.

**Figure 3:**
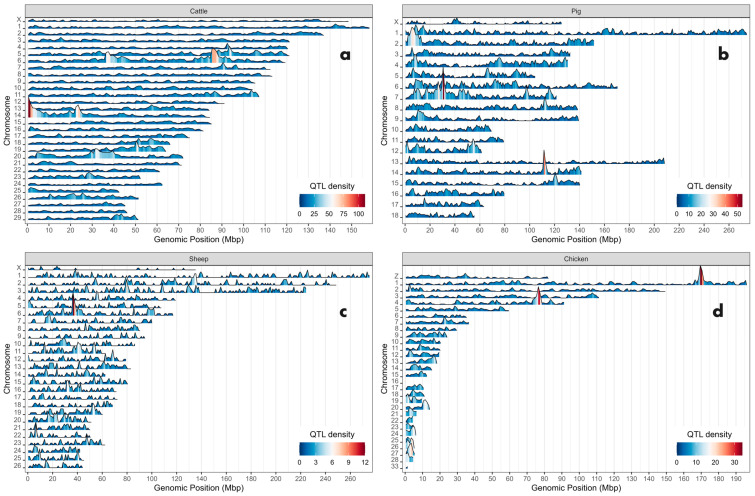
Karyogram of QTL density in four livestock genomes. (**a**) Cattle, (**b**) pigs, (**c**) sheep, and (**d**) chickens QTLome. The color gradient represents QTL density, with a shift towards red indicating genomic regions with the highest QTL density on each chromosome.

To understand the distribution of QTL across the studied genomes, their positions along the chromosomes were analyzed and compared with the distribution of genes and SNP genetic variations using karyograms. While Figure 1 illustrates the karyogram of the bovine QTLome, supplementary figures (Figures S1–S3) present the QTLomes of the other species. The graphs reveal that most chromosomes contain QTL, but their distribution varies within each chromosome, showing regions of both lower and higher QTL densities. On average, the density of QTL (per Mbp) displayed median values of 10 for bovine, 4 for pigs, 1 for sheep, and 3 for chickens. Notably, chromosomes BTA20, SSC7, OAR20, and GGA16 exhibited the highest QTL densities, with medians of 15.5, 7, 2, and 19 QTL per Mbp, respectively (Figure 2). In contrast, the sexual chromosomes (X or Z) in all four species had the lowest densities, with BTA X in cattle showing 4.32 QTL per Mbp, and fewer than 2 QTL per Mbp in the other species. The genomic regions with the highest numbers of unique QTL were located at 0.5 Mbp on BTA14 (*n* = 111), 30.5 Mbp on SSC7 (*n* = 54), 36.5 Mbp on OAR6 (*n* = 12), and 169.5 Mbp on GGA1 (*n* = 34). These regions, along with other high-QTL-density areas, are visible in the linear karyograms (Figure 3). For example, in the bovine genome, segments of chromosomes 5, 6, 19, and 20 are noteworthy due to their pronounced peaks with high QTL abundance. Considering the location and span of the QTL, the percentage of the genome covered by QTL was 87.30% for bovine, 66.22% for pigs, 20.75% for sheep, and 57.25% for chickens.

The graphical analysis of the QTLomes also revealed an apparent correlation between the distribution of QTL and the distribution of genes, as illustrated in Figure 1 and Figures S1–S3. It was observed that as gene density fluctuated along the chromosomes, QTL density followed a similar trend. Additionally, in certain chromosomal regions, the increase in QTL density coincided with the presence of major genes. For example, in bovine, chromosomes 14, 19, and 20 displayed higher QTL densities, which corresponded with the locations of major genes. Similarly, in pigs, chromosomes 7, 9, 12, and 15 displayed this pattern, as did chromosomes 2, 6, and 11 in sheep, and 1, 2, 7, 12, 13, 20, and 17 in chickens. In contrast, the distribution of genetic variations in the SNPdb aligned more closely with gene distribution than with QTL. Microarray SNP, however, displayed a more uniform distribution and did not show a strong association with either QTL or gene locations. Further statistical analyses to assess these patterns are detailed in the sections below.

### 3.2. Distribution of QTL and Their Relationship with Gene Count and Chromosome Size

**Figure 4:**
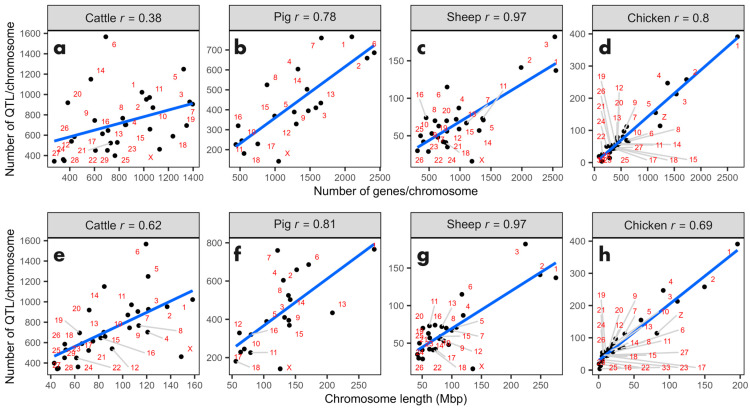
Relationship between the number of QTL, gene count, and chromosome size in four livestock genomes. Scatter plots for (**a**,**e**) cattle, (**b**,**f**) pigs, (**c**,**g**) sheep, and (**d**,**h**) chickens. Each point on the graph represents a chromosome, illustrating the correlation between the number of QTL and both gene count and chromosome size across the different species. Blue line represents the linear regression line. Chromosomes are indicated by numbers and gray lines.

To investigate the relationship between the number of QTL, gene count, and chromosome size, correlation analyses (Pearson) and simple linear regression were conducted. The aim was to determine the extent to which the distribution of QTL could be explained by these variables. At the genomic level, positive linear relationships were identified in all four analyzed genomes (Figure 4). The correlation values between the number of QTL and the number of genes ($r_{QTL,\;genes}$) as well as between QTL and chromosome size ($r_{QTL,\;chr\;size}$) were moderate to strong and statistically significant (*p* < 0.05).

In descending order of magnitude, the highest correlation was observed in the chicken genome ($r_{QTL,\;genes}$ = 0.97, $r_{QTL,\;chr\;size}$ = 0.97). Following this, the sheep genome exhibited strong correlations ($r_{QTL,\;genes}$ = 0.78, $r_{QTL,\;chr\;size}$ = 0.81), while the pig genome showed moderate correlations ($r_{QTL,\;genes}$ = 0.80, $r_{QTL,\;chr\;size}$ = 0.69). The bovine genome displayed relatively lower correlations ($r_{QTL,\;genes}$ = 0.38, $r_{QTL,\;chr\;size}$ = 0.62). A similar pattern of correlation was found between the number of genes and chromosome size ($r_{QTL,\;chr\;size}$ = 0.98, 0.86, 0.72, and 0.52, respectively, as shown in Figure S4). In general, these positive correlations suggest that as chromosomes increase in size or gene content, they tend to contain a greater number of QTL. In essence, both variables serve as reliable indicators of the expected number of QTL on each chromosome.

### 3.3. Correlation Between QTL and Gene Density at the Chromosome Level

**Figure 5:**
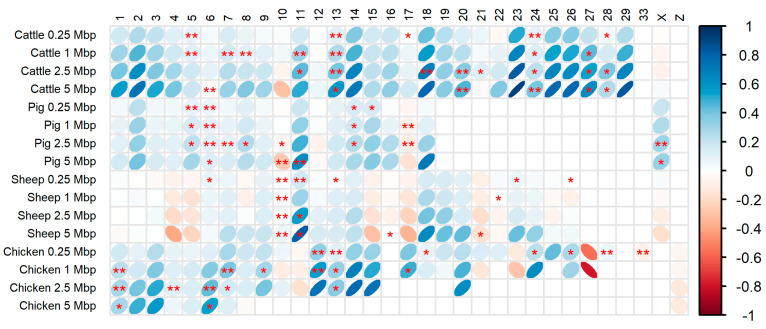
Correlation matrix between QTL density and gene density across chromosomes. Correlations are displayed for each chromosome at varying resolution scales (genomic windows of 0.25, 1, 2.5, and 5 Mbp). Only chromosomes with 10 or more observations are included. The significance of the correlations is indicated by asterisks: * = 0.05 and ** = 0.01.

We conducted an investigation to assess whether gene distribution influences the distribution of QTL within each chromosome. The number of annotations was quantified in genomic windows of 0.25, 1, 2.5, and 5 Mbp, and their correlations ($r_{0.25},\;r_{1},\;r_{2.5},\;r_{5}$) were evaluated. Figure 5 depicts the relationship between these variables for each chromosome. Upon analyzing the scatter plots, we observed a variety of relationships, including both positive and negative linear correlations of different magnitudes, as well as instances where no clear correlation between QTL and gene density was evident (Table S3). In the 0.25 Mbp windows, most chromosomes showed low or no correlation between QTL and gene density. However, as the window size increased (1, 2.5, and 5 Mbp), the correlation strength generally increased, while the overall pattern of association remained consistent.

Referring to the results obtained using 1 Mbp windows, the highest correlation between gene density and QTL density was observed in the bovine genome, followed by chicken, pig, and sheep genomes, with mean correlation values of ${\bar{r}}_{1}$ = 0.25, 0.19, 0.12, and 0.03, respectively. The correlation values varied widely across chromosomes, ranging from −0.04 (BTA X) to 0.66 (BTA 23) in cattle; −0.06 (SSC 17) to 0.33 (SSC 11) in pigs; −0.15 (OAR 5) to 0.31 (OAR 11) in sheep; and −0.78 (GGA 27) to 0.68 (GGA 14) in chickens. Some chromosomes exhibited particularly low $r_{1}$ correlation coefficients, such as BTA 6, 10, 12, 17, 21, 22, and X in bovine; SSC 1, 3, 4, 6, 7, 9, 10, 12, 16, and 17 in pigs; OAR 1, 2, 3, 6, 8, 10, 12, 16, 21 to 26, and X in sheep; and GGA 11, 19, and Z in chickens (Table S3). It is important to note that not all correlation values at the chromosomal level were statistically significant, with only seven significant correlations in bovine, four in pigs, two in sheep, and six in chickens (Figure 5).

When analyzing the correlation between SNP and QTL density, similar correlation values were observed (Figure S5 and Table S4), with ${\bar{r}}_{1}$ increasing only in the pig and chicken genomes (${\bar{r}}_{1}$ = 0.36 and 0.33, respectively). The ${\bar{r}}_{1}$ correlation between the two predictors, gene and SNP density, also varied across genomes (Figure S6 and Table S5), ranging from positive and moderate in the bovine genome (${\bar{r}}_{1}$ = 0.54) to low and negative in the sheep genome (${\bar{r}}_{1}$ = −0.38).

### 3.4. QTL Enrichment Analysis by Chromosome

**Figure 6:**
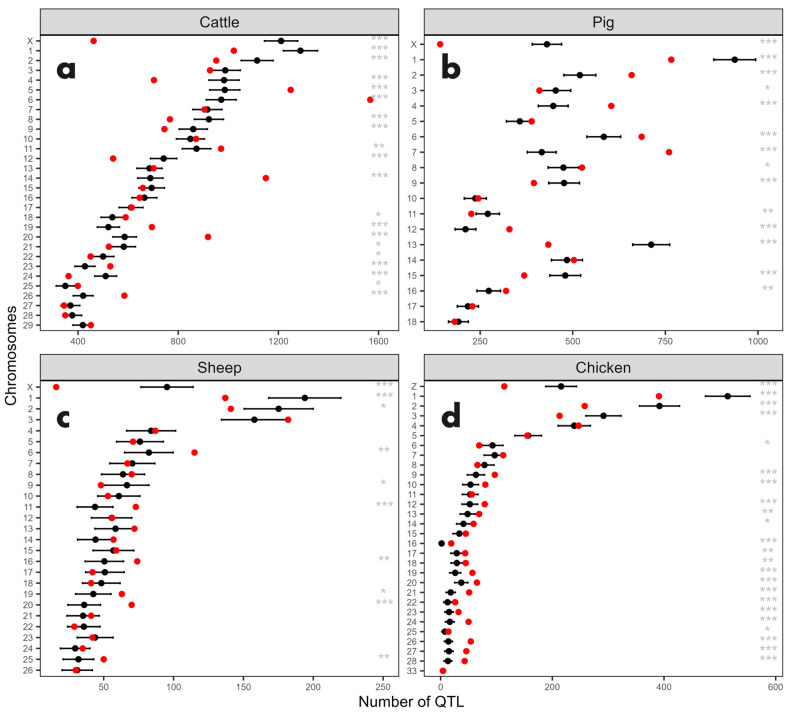
Expected and observed number of QTL in the enrichment analysis by chromosome. Plots for (**a**) bovine, (**b**) pig, (**c**) sheep, and (**d**) chicken chromosomes. Red dots represent the observed number of QTL, while black dots indicate the expected number of QTL, accompanied by the 95% confidence interval. Asterisks denote the significance level of the binomial test, with * indicating *p* < 0.05, ** indicating *p* < 0.01, and *** indicating *p* < 0.001.

Based on the results, further analyses were conducted to identify chromosomes statistically enriched or depleted in QTL. The enrichment analysis showed that the number of QTL observed in each chromosome closely matched the expected values, particularly in sheep and pig chromosomes as shown in Figure 6 and Table S6. In contrast, several bovine and pig chromosomes exhibited QTL counts significantly different from expectations. When scatter plots of the number of QTL versus chromosome size or gene content were analyzed (Figure 6), certain chromosomes stood out from the main distribution, such as bovine chromosomes 5, 6, 14, and 20, pig chromosome 7, sheep chromosome 3, and the X chromosome across species (Figure 6a).

The analysis identified the following chromosomes as significantly enriched (*p* < 0.05) of QTL: 5, 6, 11, 14, 19, 20, 23, 25, and 26 in the bovine genome; 2, 4, 6, 7, 8, 12, and 16 in the pig genome; 6, 11, 16, 19, 20, and 25 in the sheep genome; and 9 to 28 (excluding 11 and 15) in the chicken genome. On the other hand, chromosomes that had significantly fewer QTL than expected included BTA 1, 2, 4, 8, 9, 12, 21, 22, 24, and X in bovine; SSC 1, 3, 9, 11, 13, 15, and X in pigs; OAR 1, 2, 9, and X in sheep; and GGA 1, 2, 3, 6, and Z in chickens.

### 3.5. Regional Analysis for Identifying Chromosomal Segments Enriched by QTL

**Figure 7:**
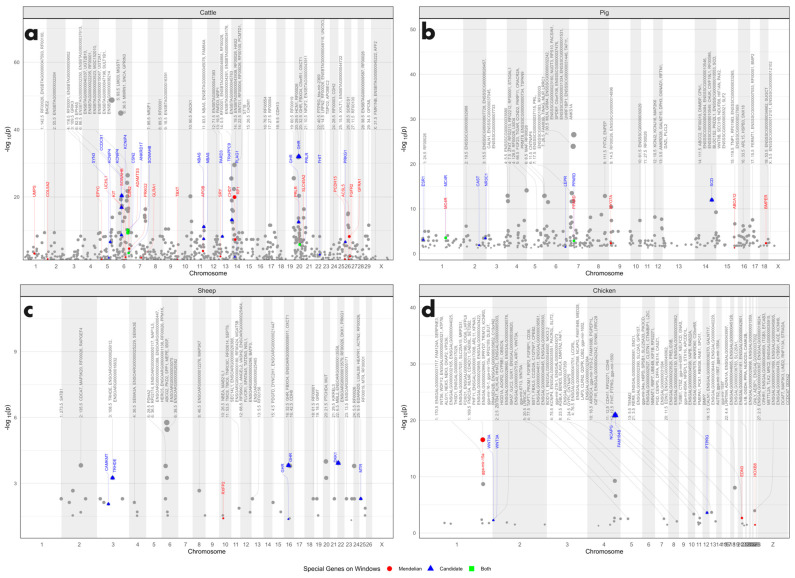
Manhattan plot of QTL enrichment analysis in four livestock genomes. (**a**) Cattle, (**b**) pig, (**c**) sheep, and (**d**) chicken QTLome. Only significant windows (*p* < 0.05) are displayed. The graph displays the genes located in the most significant genomic regions of each chromosome. All points (including gray, red, blue, and green points) indicate significantly enriched genomic windows. Red points represent genomic windows containing major genes, blue points indicate windows with candidate genes, and green points represent windows containing both major and candidate genes.

Using gene density as the success probability in a binomial enrichment analysis, 1142 genomic windows were identified as significantly enriched in QTL across the four studied genomes (*p* < 0.05, Table S7). In the bovine genome, 699 windows were found to be QTL-enriched, with the most significant peaks occurring on chromosomes 5, 6, 7, 10, 14, 20, 26, and 29 (Figure 7). These regions harbor major genes such as *KIT*, casein beta (*CSN2*), sex determining region Y (*SRY*), and *PRLR*, as well as candidate genes like *PLAG1* zinc finger (*PLAG1*) and *GHR*. In the pig genome, 355 QTL-enriched windows were detected, with the highest peaks on chromosomes 4, 5, 7, 8, and 14 (Figure S7). Major genes in these regions included melanocortin 4 receptor (*MC4R*), peroxisome proliferator activated receptor delta (*PPARD*), myosin VIIA (*MYO7A*), ATP binding cassette subfamily A member 12 (*ABCA12*), and BMP binding endothelial regulator (*BMPER*), while candidate genes included *CAST*, *LEPR*, and *SCD*. In the sheep genome, 50 QTL-enriched windows were found, with the most significant peak located on chromosome 6 (Figure S8). Notably, relaxin family peptide receptor 2 (*RXFP2*) was identified on chromosome 10, while candidate genes included calmodulin-lysine *n*-methyltransferase (*CAMKMT*), thyrotropin-releasing hormone-degrading enzyme (*TRHDE*), *GHR*, Dickkopf WNT signaling pathway inhibitor 1 (*DKK1*), and 5-methyltetrahydrofolate-homocysteine methyltransferase (*MTR*). In the chicken genome, 38 QTL-enriched segments were identified, with significant peaks on chromosomes 1, 4, and 19. The major genes in these windows included microRNA 15a (*GGA-MIR−15A*), endothelin 3 (*EDN3*), and homeobox B8 (*HOXB8*), while candidate genes included Wnt family member 3A (*WNT3A*), Wnt family member 9A (*WNT9A*), non-SMC condensin I complex subunit G (*NCAPG*), family with sequence similarity 184 member B (*FAM184B*), and protein tyrosine phosphatase receptor type G (*PTPRG*).

**Figure 8:**
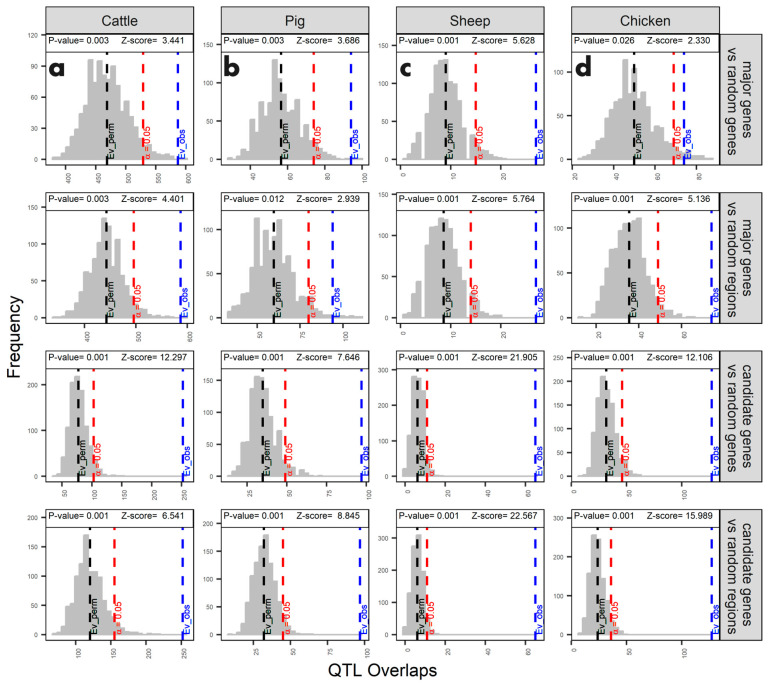
Distribution of overlaps between QTL and major or candidate genes. Analysis for (**a**) bovine, (**b**) pig, (**c**) sheep, and (**d**) chicken genomes. For each enrichment analysis, the number of overlaps between QTL and major or candidate genes was compared to overlaps with randomly selected genes or genomic segments. The right-tailed hypothesis test assesses whether the observed number of overlaps (QTL with genes of interest) is significantly greater than the average number of overlaps obtained through resampling (QTL with randomly selected genes or segments).

A resampling enrichment analysis revealed that chromosomal windows containing major or candidate genes had significantly more QTL (*p* < 0.05) compared to randomly selected genes or genomic segments (Figure 8). Notably, the major genes with the highest QTL (unique) overlap were *CSN2* in bovine (20 QTL); protein kinase AMP-activated non-catalytic subunit gamma 3 (*PRKAG3*) in pigs (8 QTL); laminin subunit gamma 2 (*LAMC2*) (3 QTL), beta-1,4-*n*-acetyl-galactosaminyltransferase 2 (*B4GALNT2*) (3 QTL), *MSTN* (3 QTL), and *RXFP2* (3 QTL) in sheep; and *GGA-MIR-15A* in chickens (12 QTL). The candidate genes with the highest QTL (unique) overlap were potassium voltage-gated channel interacting protein 4 (*KCNIP4*) in bovine (28 QTL), estrogen receptor 1 (*ESR1*) in pigs (9 QTL), *GHR* in sheep (6 QTL), and *FAM184B* in chickens (12 QTL).

### 3.6. Regional Analysis for Identifying Chromosomal Segments Enriched by Trait-Specific QTL

**Figure 9:**
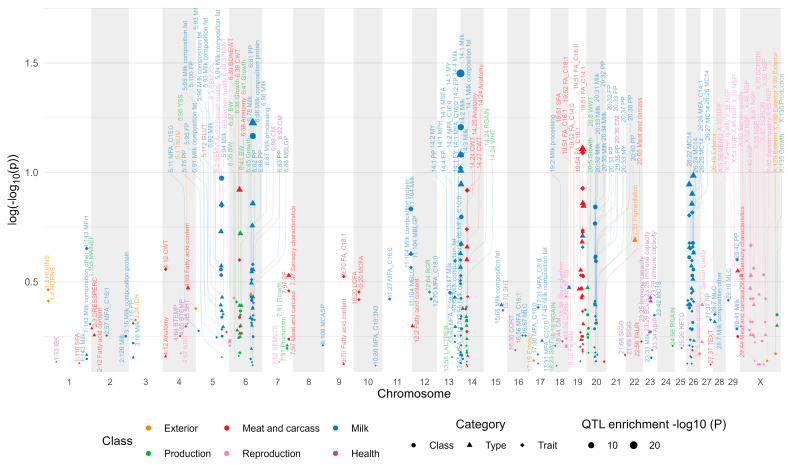
Manhattan plot of QTL enrichment analysis for bovine phenotypic categories. Only significant genomic windows (*p* < 0.05) are displayed. The graph displays the genes located in the most significant genomic regions of each chromosome. For the purposes of graph reduction, the Y-axis represents the logarithm of the negative logarithm (base 10) of the *p*-values. The traits and their abbreviations are as follows: 5HT: Serotonin level, ABIR: Antibody_mediated immune response, BLVS: Bovine leukemia virus susceptibility, BONEWT: Bone weight, BTEMP: Body temperature, BW: Body weight (birth), CABIL: Calving ability, CM: Clinical mastitis, CORT: Blood cortisol level, CWT: Carcass weight, DRESSPERC: Dressing percentage, FA_C14:0: Myristic acid content, FA_C14:1: Myristoleic acid content, FA_C16:0: Palmitic acid content, FA_C16:1: Palmitoleic acid content, FA_C18:1: Oleic acid content, FP: Milk fat percentage, FY: Milk fat yield, GLUT: Glutamate level, HORNS: Presence of horns, HPG: Heifer pregnancy, IBK: Infectious bovine keratoconjunctivitis susceptibility, IDH: Interdigital hyperplasia, KETO: Ketosis, LACTPER: Lactation persistency, LCFA: Long_chain fatty acid content, MAINEF: Maintenance efficiency, MBLG: Milk beta_lactoglobulin protein content, MBLGP: Milk beta_lactoglobulin percentage, MC10: Milk c10 index, MC14: Milk c14 index, MC18: Milk c18 index, MCASP: Milk casein percentage, MCFA: Medium_chain fatty acid content, MFA_C10:0: Milk capric acid content, MFA_C12:0: Milk lauric acid content, MFA_C14:0: Milk myristic acid content, MFA_C14:1: Milk myristoleic acid content, MFA_C15:0: Milk pentadecylic acid content, MFA_C16:0: Milk palmitic acid content, MFA_C16:1: Milk palmitoleic acid content, MFA_C17:0: Milk margaric acid content, MFA_C18:0: Milk stearic acid content, MFA_C18:1: Milk oleic acid content, MFA_C18:3N3: Milk linolenic acid content, MFA_C8:0: Milk caprylic acid content, MKCAS: Milk kappa_casein content, MLC: Milk lactose content, MPH: Milk phosphorus content, MSFA: Milk saturated fatty acid content, MUFA: Monounsaturated fatty acid content, MY: Milk yield, NRR: Non_return rate, NSP: Percentage normal sperm, PP: Milk protein percentage, RGAIN: Residual gain, RGR: Relative growth rate, SB: Stillbirth, SCRCIR: Scrotal circumference, SEMENQ: Sperm counts, SEMVOL: Semen volume, SF: Shear force, SFA: Saturated fatty acid content, SIGG: Immunoglobulin g level, SPMINT: Sperm plasma membrane integrity, SWT: Body weight (slaughter), TAUR: Muscle taurine content, TEXT: Meat texture, TNUM: Teat number, WHT: Withers height, WWT: Body weight (weaning), YSS: Young stock survival.

The hypergeometric test identified 287 genomic windows statistically enriched by QTL associated with important traits across the four studied genomes (*p* < 0.05, Table S8). In the bovine genome, 187 windows were enriched with QTL linked to 92 phenotypes (Figure 9 and Table S8). The number of significant associations per phenotypic category was 62 for classes, 113 for types, and 188 for traits. Specifically, the significant windows per phenotypic class were distributed as follows: 11 for exterior traits, 20 for health, 62 for meat and carcass, 185 for milk, 44 for production, and 41 for reproduction. The most significant peaks occurred at 5:94, 6:86, 14:1–3, 20:33, 26:21–22, and 21:26 Mbp, with windows predominantly enriched by QTL related to milk traits. Other important peaks were located at 6:38, 14:25, and 19:51–52 Mbp, which were associated with meat and carcass traits. Additionally, the X chromosome exhibited an enrichment of QTL associated mainly with reproduction traits. Two windows, located at 6:38 and 14:24 Mbp, were enriched by QTL related to both production and meat and carcass traits.

In the pig genome, 68 genomic windows were statistically enriched by QTL associated with 49 phenotypes (Figure S7 and Table S8). The number of significant associations per phenotypic category was seven for classes, 29 for types, and 66 for traits. Specifically, the significant windows per phenotypic class were distributed as follows: 18 for exterior traits, 18 for health, 69 for meat and carcass, five for production, and two for reproduction. The most significant peaks were located at 7:47, 8:112, 12:1–2, 14:112–113, and 16:39–41 Mbp, with genomic windows predominantly enriched by QTL related to meat and carcass traits.

Within the sheep genome, 15 windows were found to be statistically enriched by QTL associated with 13 distinct phenotypes (Figure S8 and Table S8). The number of significant associations per phenotypic category was two for classes, nine for types, and 10 for traits. Specifically, the significant windows per phenotypic class were distributed as follows: six for exterior traits, four for health, five for meat and carcass, two for milk, one for production, and three for wool. Notable significant windows include those associated with exterior traits, such as horn number (132–134 and 241 Mbp on OAR 2), meat and carcass traits like loin yield (2:129 Mbp) and meat color (12:1 Mbp), health traits such as mastitis (2:207–208 and 16:36 Mbp) and fecal egg count (14:49 Mbp), wool traits (16:26 and 25:10 Mbp), milk traits (13:61–62 Mbp), and animal stature (9:32 Mbp).

In the chicken genome, 17 windows were statistically enriched by QTL associated with 10 distinct phenotypes (Figure S9 and Table S8). The number of significant associations per phenotypic category included two for classes, 10 for types, and eight for traits, with windows corresponding to the exterior, physiology, production, and reproduction classes. These significant windows were associated with productive traits such as egg quality (1:165–166 Mbp), growth (1:170, 2:3, 4:77–79, and Z:12–13 Mbp), and body weight (4:78, 8:2–3, 12:13–14 Mbp). Additionally, other genomic regions exhibited enrichment of QTL for reproductive (1:174–175 Mbp), exterior (4:1–2 Mbp), and blood potassium level (12:18 Mbp) traits.

## 4. Discussion

In recent years, genomic studies on major domestic species to identify causal regions for numerous economically important traits have grown significantly. This has led to the expansion of large databases containing QTL information. With this growing data availability, the QTLomes of bovine, pigs, sheep, and chickens were analyzed to address several research questions: (1) How are QTL distributed across the genome? (2) What is the relationship between genome size and the number of QTL? (3) Are there genomic regions that contain more QTL than expected? (4) Where are the regions with high QTL concentrations for specific traits?

### 4.1. QTL Distribution Is Related to Gene Density and Chromosome Size

Our initial findings in the present study revealed that both chromosome size and gene count are strong predictors of the number of QTL on a chromosome. We found moderate to strong positive correlations between the number of QTL and gene content ($r_{QTL,\;genes}$) or chromosome size ($r_{QTL,\;chr\;size}$). These results align with findings from other studies on both animals and plants. For instance, ref. [19] reported correlations of 0.91 for gene count ($r_{QTL,\;genes}$) and 0.85 for chromosome size ($r_{QTL,\;chr\;size}$) in the Atlantic salmon QTLome. Similarly, ref. [19] found a correlation of 0.94 between reported QTL and chromosome length ($r_{QTL,\;chr\;size}$) in the maize genome. This trend was consistent across all four species in our study. In bovine, however, the correlations between QTL and both gene count and chromosome size were lower ($r_{QTL,\;genes}$ = 0.38; $r_{QTL,\;chr\;size}$ = 0.62), likely due to the large number of QTL on certain outlier chromosomes such as BTA 6 and 14. Excluding these chromosomes improved the correlations, with $r_{QTL,\;genes}$ rising to 0.60 and $r_{QTL,\;chr\;size}$ to 0.69. It is worth noting that regions on BTA 6 and 14 have been frequently reported to harbor QTL for various traits [33,34,35], contributing to the overrepresentation of these chromosomes in the QTL database.

### 4.2. QTL Distribution in Sex Chromosomes

Interestingly, sex chromosomes across the four species contained fewer QTL than expected. This discrepancy can be attributed to the fact that many genetic mapping studies exclude the genotypic information of sex chromosomes, as these loci follow a different inheritance pattern, leading to a predominant focus on autosomes [36,37]. In cattle, for instance, annotations for BTA X were sourced from only 60 studies, compared to an average of 177 studies for other chromosomes. Additionally, no QTL have been reported on the Y chromosome in the three mammalian species or on chromosomes 30–32 and the W chromosome in chicken genome. Similarly, no QTL were found in mitochondrial chromosomes. These findings suggest a need for more research focused on locating QTL on sex chromosomes, which constitute a significant portion of the genome. In cattle alone, the X chromosome represents 5.59% of the genome and contains 1128 genes (4.58%), making it one of the largest and most gene-rich chromosomes (https://www.ensembl.org/index.html, accessed on 26 April 2023).

### 4.3. Correlation Between QTL and Gene Density in Genomic Windows

The second major finding of this study was that the correlation between QTL and gene density in genomic windows (0.25, 1, 2.5, and 5 Mbp) varies across chromosomes. For example, in cattle, positive correlations were observed between gene density and QTL, except for chromosomes 10, 21, 22, and X. Overall, 57% of chromosomes in the four analyzed species exhibited positive correlations between the number of QTL and gene density (${\bar{r}}_{1}$ > 0.1). This suggests that while gene density may be a useful predictor of QTL distribution in some regions, other factors, such as the presence of major genes, pleiotropic regions, and linkage disequilibrium, likely also play important roles in shaping the observed patterns of QTL distribution. The significance of these findings lies in the observation that gene density can serve as an indicator of the presence of QTL, as demonstrated in BTA 23 ($r_{1}$ = 0.66) of cattle and GGA 14 ($r_{1}$ = 0.68) of chickens. While SNP density also showed a significant correlation with QTL density, it is important to note that the SNP analyzed represent only a fraction of the total genetic variation in each genome. Therefore, gene density is a more feasible predictor for the distribution of QTL at the chromosomal level.

Previous studies have similarly linked the location of QTL to gene distribution in chromosomes. For example, ref. [19] reported a correlation of 0.34 between gene density and QTL density in 5 Mbp windows for Atlantic salmon (${\bar{r}}_{5}$), while the bovine genome showed an ${\bar{r}}_{5}$ of 0.41. The authors of [10] also observed that chromosomal segments containing QTL were associated with higher gene density in cattle, though the study was limited to 597 QTL, making it difficult to quantify the degree of association through correlation. With the current bovine QTL dataset, which includes 21,748 unique QTL, we were able to quantify the relationship between QTL and gene density more accurately, finding higher correlation values than those reported in other species.

Additionally, the correlation between QTL and gene density improved as databases contained more annotations. For instance, the bovine QTLome showed ${\bar{r}}_{1}$ = 0.25, while the ovine QTLome with only 1831 unique QTL presented ${\bar{r}}_{1}$ = 0.03. The average correlation coefficients ($\bar{r}$) also increased as the resolution scale of genomic windows increased. In the bovine genome, the average correlation $\bar{r}$ in windows of 0.25, 1, 2.5, and 5 Mbp was 0.16, 0.25, 0.34, and 0.41, respectively, a pattern observed in other species as well. However, certain chromosomes exhibited low or negative correlations, likely influenced by QTL enrichment or depletion. For example, bovine chromosome 6, with an $r_{1}$ = 0.06, showed significant QTL enrichment (*p* = 2.73 × 10^−71^), while the X chromosome, with an $r_{1}$ = −0.04, showed significant depletion (*p* = 3.36 × 10^−138^). These were the most significant chromosomes in the two-tailed binomial hypothesis test of the bovine genome.

The high correlation between QTL and gene density was unexpected because genetic mapping studies typically do not map QTL directly to causal genes. It is crucial to note that current genetic mapping strategies primarily rely on genotyping microarrays designed based on genetic variations selected according to allele frequency and segregation in different populations [38,39]. Therefore, it is not anticipated that QTL will align precisely with causal genes, at least when utilizing the commercially developed microarrays.

As mentioned, while QTL are distributed across chromosomes, certain genomic regions show an enrichment of QTL. An enrichment analysis of the entire QTLome identified a total of 699, 355, 50, and 38 statistically significant windows in the cattle, pig, sheep, and chicken genomes, respectively. This analysis, combined with the bootstrap enrichment test on genes of interest, revealed that QTL enrichment in some genomic windows correlates with the presence of major and candidate genes. These genes are frequently the focus of studies aimed at identifying QTL and genetic variations that are valuable for animal breeding [40,41]. This concentration of interest is reflected in the number of reports in the AnimalQTLdb database. Additionally, GWAS often report QTL at similar genomic locations, leading to certain regions being overrepresented [35]. In all four QTLomes analyzed, a strong correlation (r > 0.73) was found between the number of QTL and the number of studies or phenotypic traits examined, suggesting that QTL density is also associated with pleiotropic regions.

Through the trait-specific QTL enrichment analysis, genomic regions associated with multiple traits were identified. Particularly in the bovine and pig QTLomes, certain chromosomes were found to contain QTL linked to traits within the same phenotypic category. For example, bovine chromosomes 5, 6, 14, 20, 21, and 26 contain QTL related to milk traits, while pig chromosomes 7, 8, 12, 14, and 16 are associated with meat and carcass traits. Some chromosomal regions have been reported to exhibit specialization toward specific phenotypic traits. For instance, ref. [24] discovered genes associated with fertility traits clustered in regions of chromosomes X, 14, and 17 in the bovine genome. Reference [22] identified at least one pleiotropic QTL every 50 Kbp in the bovine genome, which had an effect on 34 productive traits in dairy cattle populations. In general, pleiotropy has been extensively studied in various genes and genomic regions across different species, with its effects being examined on traits from multiple phenotypic categories. For example, ref. [20] reported pleiotropic effects from 28 QTL located on chromosomes 3, 5, 6, 7, 14, 20, and 29, affecting 32 traits including growth, body composition, feed intake, and reproduction in beef cattle. Additionally, ref. [21] described the pleiotropic influence of several genes, including *DGAT1* (BTA 14:1.8 Mbp), microsomal glutathione S-transferase 1 (*MGST1*) (BTA 5:93 Mbp), progestagen associated endometrial protein (*PAEP*) (BTA 11:103 Mbp), glycerol-3-phosphate acyltransferase 4 (*GPAT4*) (BTA 27:36 Mbp), *CSN2* (6:87), mucin 1, cell surface associated (*MUC1*) (BTA 3:15.6 Mbp), *GHR* (BTA 20:31.2 Mbp), syndecan 2 (*SDC2*) (BTA 14:70 Mbp), calpain 1 (*CAPN1*) (BTA 29:44 Mbp), and *CAST* (BTA 7:96 Mbp), on traits such as protein, fat, and milk production in dairy cattle. These findings highlight the multifaceted role that certain genomic regions and genes play across a range of traits in livestock.

### 4.4. Research Perspectives

The genomic regions enriched by QTL identified in this study could serve as a basis for prioritizing candidate genes or causal variants, as demonstrated by the following examples. Ref. [14] used fine mapping to resequence the 128–136 Mbp region of pig chromosome 15, chosen for its high density of QTL linked to multiple productive traits. They discovered SNP in the tensin 1 (*TNS1*), vilin 1 (*VIL1*), and ubiquitin specific peptidase 37 (*USP37*) genes, which showed allele frequency differences between two pig breeds phenotypically distinct for meat quality, fat content, and growth. Similarly, ref. [12] used meta-analysis to refine the number and confidence intervals of 75 QTL associated with key traits in pigs. For fat thickness and average daily gain, they highlighted four meta-QTL regions, coinciding with the location of the FAT atypical cadherin 1 (*FAT1*) gene, and identified 15 additional regions containing QTL for other traits and phenotypic categories. In another meta-analysis, ref. [11] focused on a genomic region of pig chromosome 1, where 21 QTL related to average daily gain had been previously reported. Their analysis suggested that these QTL were likely due to 6–8 distinct QTL. The authors of [42] proposed a gene prioritization strategy for productive traits based on the association of genes within QTL regions with biological processes and identifying those overrepresented in relevant functional categories. The authors of [43] used a similar approach to analyze 201 QTL from 10 GWAS, identifying a KEGG pathway relevant to residual feed intake (RFI) in cattle and prioritizing the methylcrotonyl-CoA carboxylase subunit 1 (*MCCC1*), aldehyde oxidase 1 (*AOX1*), and propionyl-CoA carboxylase subunit alpha (*PCCA*) genes. Other integrative strategies have been proposed by [17,25,44], who used systems biology approaches and gene expression data for gene prioritization within QTL.

Alternative strategies for enrichment analysis and QTL clustering into phenotypic categories may yield different results than those obtained in this study. For instance, ref. [45] proposed a method for identifying QTL-enriched regions (QTL hotspots) by comparing an expected QTL matrix (EQF) against a theoretical uniform distribution. The research of [46] improved upon this by grouping QTL into genetically correlated trait categories rather than using arbitrary QTL clustering, thereby reducing the EQF matrix and false positive rates and avoiding the identification of spurious genomic regions. Using this approach, ref. [46] identified more than 100 statistically enriched QTL regions in the rice genome. In this study, we used the phenotypic categories defined in the AnimalQTLdb database, which classifies QTL into classes, types, and traits. Therefore, the results of our enrichment analysis based on these phenotypic categories—especially classes and types—should be interpreted with caution, as these groupings may not necessarily include genetically or phenotypically correlated variables.

Since the AnimalQTLdb contains QTL for a wide array of economically important phenotypes, it would be highly beneficial to evaluate genomic correlations between traits based on the positions of their QTL. This could help in identifying genomic regions that are shared across different productive traits [47]. Such an analysis would allow the quantification of genomic similarity between traits and help delineate regions with possible pleiotropic effects. A spatial correlation analysis [48] between classes, types, or traits could measure the degree of phenotypic trait similarity based on the location of their QTL. It is expected that biologically related traits would exhibit similarities in their QTL distribution. For instance, ref. [13] examined the distribution of QTL for growth and immunity traits in pigs, aiming to locate genomic regions that influence both. They identified 405 genes located in regions shared between growth and immunity QTL, as well as 743 SNP with allele frequency differences across two pig breeds.

In cases where phenotypic traits have relatively few QTL, conducting an enrichment analysis may not be feasible. Instead, identifying overlaps between different studies could provide a better strategy for recognizing important genes and QTL [49]. By leveraging the data from these studies, researchers can identify overlapping or validated QTL across multiple investigations, leading to a more refined understanding of key genomic regions. This strategy could also be applied to analyze the QTLome of pigs and chickens, as their QTL are derived from only 1200 scientific studies. Identifying such validated QTL would be particularly valuable for advancing genomic research in less-studied domestic species, such as ducks [50] and turkeys [51], where fewer QTL resources exist. Notably, the only poultry QTLome currently reported in the AnimalQTLdb is that of chicken, making such comparative approaches essential for broader genomic understanding. A similar approach has been successfully applied to study pig QTL related to obesity in human genetics. This approach could enhance the discovery of orthologous genes and facilitate cross-species comparisons, offering insights into shared genetic mechanisms underlying important traits.

The functional relevance of the genes and QTL identified in this study is not discussed due to the wide variety of phenotypes represented in the database. Therefore, it would be essential to conduct functional analyses specifically for each trait category. Extensive reviews on the biological importance of some genes and QTL identified in this work can be found in sources such as the following: [8,16,52] for milk QTL; [9] for fertility-associated QTL; and [53] for growth, meat, and carcass QTL in cattle. For other species, key reference sources include the following: [54] for reproductive traits in pigs; [55] for production and reproduction traits in sheep; [56] for wool traits; and [57] for behavioral traits, among others.

## 5. Conclusions

By characterizing the distribution and density of QTL across the genomes of four livestock species, this study identified notable variation in the number of QTL per chromosome (274–1396 for bovine, 440–2417 for pig, 312–2550 for sheep, and 87–2688 for chicken), along with a non-uniform distribution within the chromosomes. Certain genomic regions were found to be enriched with QTL (699, 355, 50, and 38 for bovine, pigs, sheep, and chickens, respectively), some of which are associated with diverse phenotypic traits. The results demonstrate that QTL reported in the AnimalQTLdb are predominantly located in gene-rich regions, as evidenced by the strong correlation between QTL distribution and gene density at both genomic and chromosomal levels. Several factors influenced QTL distribution and density, including genome size (both number of genes and chromosome length), the number of annotations, study window size, the number of studies, and the presence of candidate and major genes. This research is one of the most comprehensive studies on the QTLome of livestock species, offering significant insights into the relationship between QTL enrichment and productive traits. The genomic regions enriched with QTL identified in this study offer a valuable basis for further investigation of related genes and genetic variants using resequencing, prioritization, or meta-analysis strategies.

Among the studied species, the bovine QTLome stands out as the most representative, with the highest number of QTL and phenotypic traits reported. This makes it a prime candidate for genomic-functional and gene prioritization studies that could facilitate fine mapping of specific QTL. Genomes with a greater number of annotations could be used to assign potential QTL in other species. For instance, comparative genomics could establish links between the association of a particular region or gene—such as in bovine—and the corresponding gene (ortholog) in buffalo or other species, facilitating faster discovery of QTL in less-studied species. In contrast, the ovine genome, with fewer QTL, represents an area of opportunity for future genetic mapping research. Overall, the findings of this study emphasize the depth of knowledge available on the QTLome of each species and highlight the potential for discovering functional information within their genomes. These insights provide a strong foundation for advancing genetic research in livestock species.

It is worth noting that one of the limitations of this study was the limited number of annotations available for some species (chicken, goat, horse, rainbow trout, and sheep). Additionally, the AnimalQTLdb database does not include all reported QTL, and in some cases, the QTL positions differ from those originally reported. We recommend carefully reviewing both the number and locations of QTL for each study, as well as their associations with the original traits. Furthermore, using alternative statistical enrichment methods and varying genomic window sizes could produce different results from those obtained in this study.

## Figures and Tables

**Table 1:** Overview of the study data by species. Values in parentheses represent data after filtering and quality control, compared to the original data from AnimalQTLdb (version 44).

Species	Classes	Types	Traits	Studies	QTL	Unique QTL
Cattle	6	37	680 (526)	1045 (585)	161,730 (37,146)	72,532 (21,648)
Pigs	5	31	699 (605)	724 (629)	32,476 (10,861)	14,950 (8172)
Sheep	7	32 (31)	272 (251)	190 (179)	3571 (2285)	2469 (1831)
Chickens	5	17	438 (267)	347 (153)	13,938 (3083)	7143 (2660)
Total	23	117 (116)	2089 (1649)	2306 (1546)	211,715 (53,375)	97,094 (34,311)

## Data Availability

The data used in this study are available on the AnimalQTLdb website (https://www.animalgenome.org/cgi-bin/QTLdb/index, accessed on 31 January 2022).

## Supplementary Materials

The following supporting information can be downloaded at: https://www.mdpi.com/article/10.3390/genes15121513/s1, Figure S1. Circular karyogram of the pig QTLome. Figure S2. Circular karyogram of the sheep QTLome. Figure S3. Circular karyogram of the chicken QTLome. Figure S4. Relationship between gene counts and chromosome size in four livestock genomes. Figure S5. Correlation matrix between QTL density and SNP density across chromosomes. Figure S6. Correlation matrix between SNP and gene density across chromosomes. Figure S7. Manhattan plot of QTL enrichment analysis for pig phenotypic categories. Figure S8. Manhattan plot of QTL enrichment analysis for sheep phenotypic categories. Figure S9. Manhattan plot of QTL enrichment analysis for chicken phenotypic categories. Table S1. Mendelian gene list used for the enrichment analysis. Table S2. Candidate gene list used for the enrichment analysis. Table S3. Pearson correlation values between QTL and gene counts across chromosomes and window sizes (0.25, 1, 2.5, and 5 Mbp). Table S4. Pearson correlation values between QTL and SNP counts across chromosomes and window sizes (0.25, 1, 2.5, and 5 Mbp). Table S5. Pearson correlation values between genes and SNP counts across chromosomes and window sizes (0.25, 1, 2.5, and 5 Mbp). Table S6. Observed versus expected QTL by chromosome for each genome. Table S7. Genome-wide enrichment results across the four studied genomes using gene density as the success probability in a binomial test. Table S8. Genome-wide enrichment results across the four studied genomes using hypergeometric tests for trait-specific analysis.
