# Supplementary material for: Exploration and Enrichment Analysis of the QTLome for Important Traits in Livestock Species
Source: Genes (Basel). 2024 Nov 26;15(12):1513. doi: 10.3390/genes15121513 (PMC11675464; doi:10.3390/genes15121513)
Supplement: Supplementary file 1 [file genes-15-01513-s001.zip › Supplementary Figures.pdf]

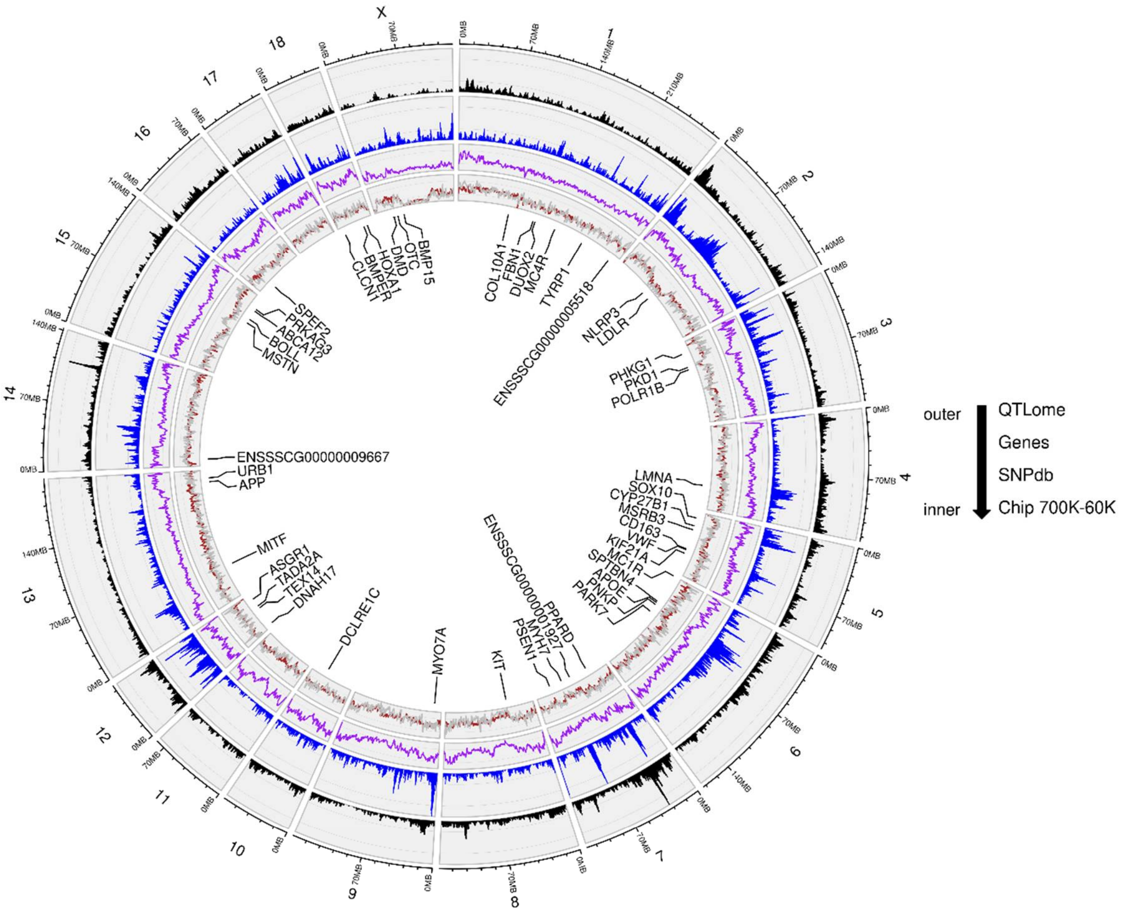

Figure S1. Circular karyogram of the pig QTLome. The first layer depicts the locations of major genes (sourced from the OMIA database), while the subsequent layers illustrate the distribution of QTL (black line), genes (blue line), and SNP from the SNPdb (purple line) and genotyping microarrays commonly used in genetic mapping studies (gray and red lines). The density of QTL, genes, and SNP is represented as the number of annotations per Mbp. Chromosomes are indicated by numbers.

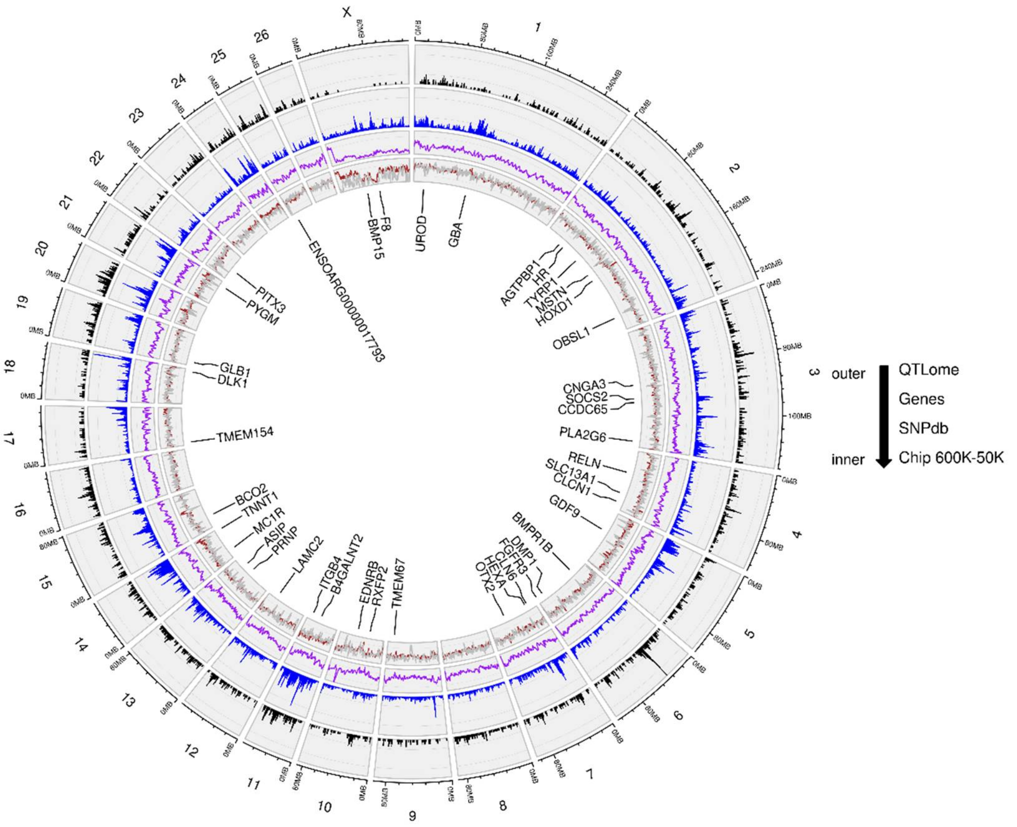

Figure S2. Circular karyogram of the sheep QTLome. The first layer depicts the locations of major genes (sourced from the OMIA database), while the subsequent layers illustrate the distribution of QTL (black line), genes (blue line), and SNP from the SNPdb (purple line) and genotyping microarrays commonly used in genetic mapping studies (gray and red lines). The density of QTL, genes, and SNP is represented as the number of annotations per Mbp. Chromosomes are indicated by numbers.

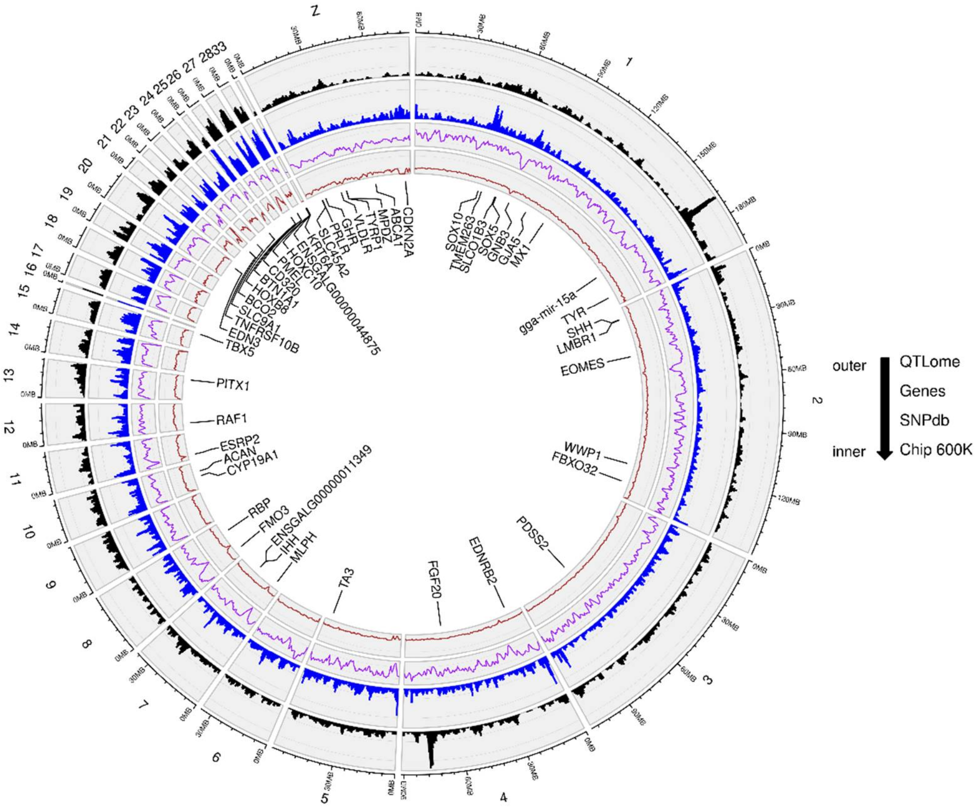

Figure S3. Circular karyogram of the chicken QTLome. The first layer depicts the locations of major genes (sourced from the OMIA database), while the subsequent layers illustrate the distribution of QTL (black line), genes (blue line), and SNP from the SNPdb (purple line) and genotyping microarrays commonly used in genetic mapping studies (red line). The density of QTL, genes, and SNP is represented as the number of annotations per Mbp. Chromosomes are indicated by numbers.

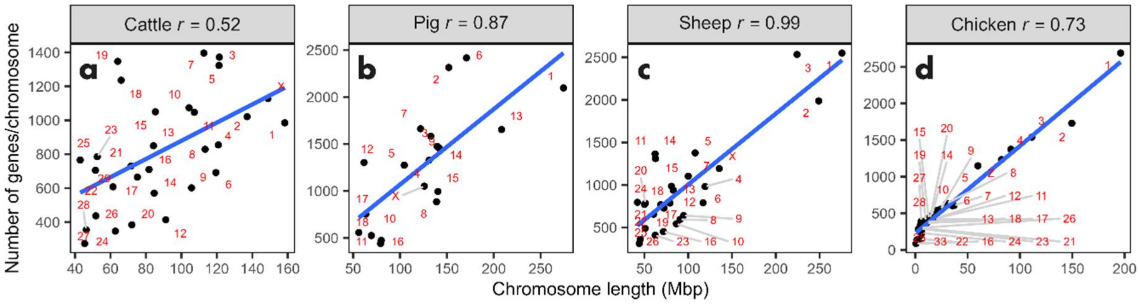

Figure S4. Relationship between gene counts and chromosome size in four livestock genomes. Scatter plots for (**a**) cattle, (**b**) pig, (**c**) sheep and (**d**) chicken. Each point on the graph represents a chromosome, illustrating the correlation between the number of genes and chromosome size across the different species. Blue line represents the linear regression line. Chromosomes are indicated by numbers and gray lines.

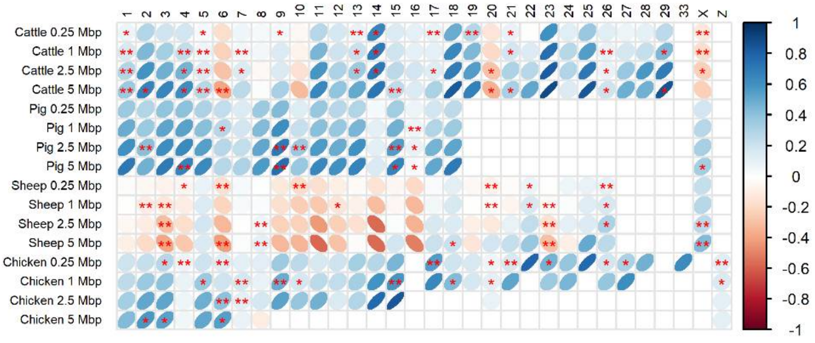

Figure S5. Correlation matrix between QTL density and SNP density across chromosomes. Correlations are displayed for each chromosome at varying resolution scales (genomic windows of 0.25, 1, 2.5, and 5 Mbp). Only chromosomes with 10 or more observations are included. The significance of the correlations is indicated by asterisks: * = 0.05 and ** = 0.01.

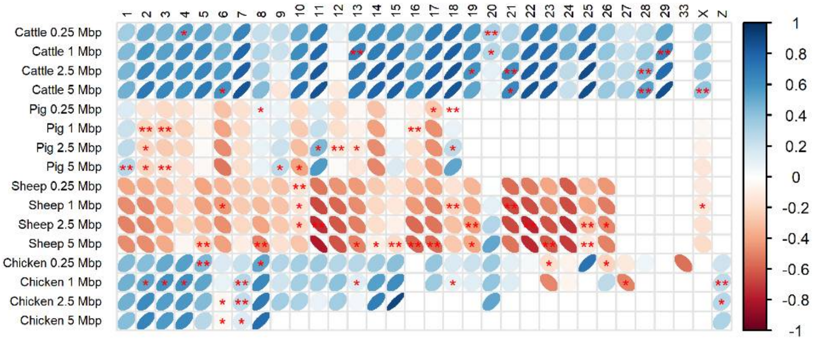

Figure S6. Correlation matrix between SNP and gene density across chromosomes. Correlations are displayed for each chromosome at varying resolution scales (genomic windows of 0.25, 1, 2.5, and 5 Mbp). Only chromosomes with 10 or more observations are included. The significance of the correlations is indicated by asterisks: * = 0.05 and ** = 0.01.

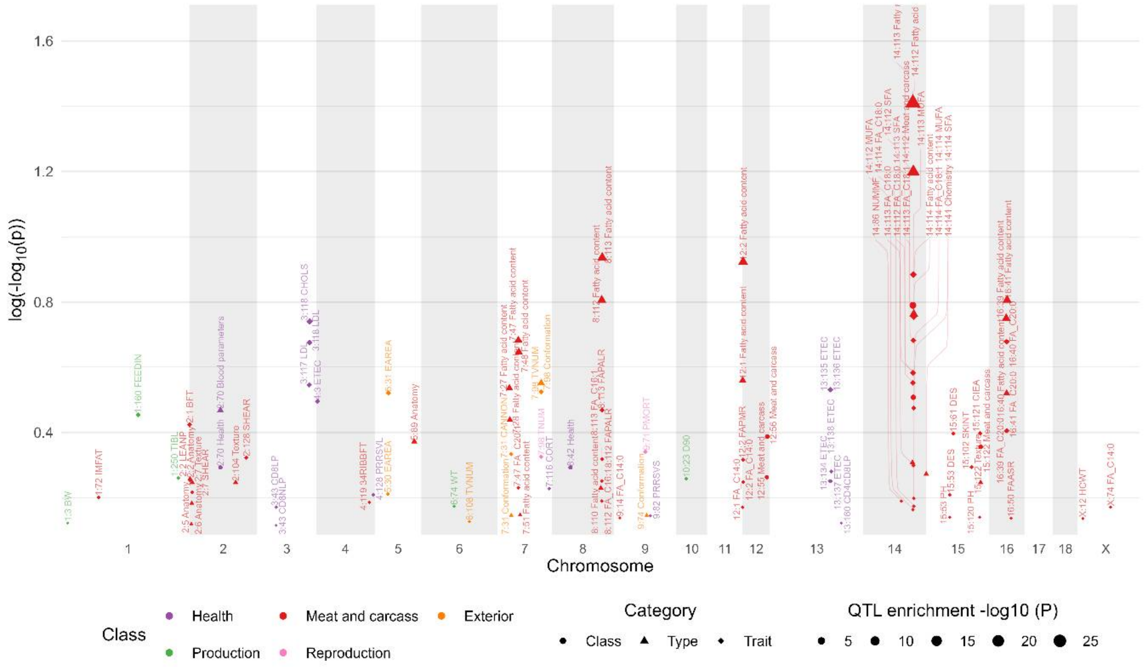

Figure S7. Manhattan plot of QTL enrichment analysis for pig phenotypic categories. Only significant genomic windows ($P < 0.05$) are displayed. The graph displays the genes located in the most significant genomic regions of each chromosome. For the purposes of graph reduction, the Y-axis represents the logarithm of the negative logarithm (base 10) of the P-values. The traits and their abbreviations are as follows: 34RIBBFT: Backfat thickness between 3rd and 4th rib, BFT: Average backfat thickness, BW: Body weight (150 days), CANNON: Cannon bone circumference, CD4CD8LP: Cd4_positive cd8_positive leukocyte percentage, CD8LP: Cd8_positive leukocyte percentage, CD8NLP: Cd8_negative leukocyte percentage, CHOLS: Cholesterol level, CIEA: Meat color a*, CORT: Cortisol level, D90: Days to 90 kg, DES: Distance from eye to end of snout, EAREA: Ear area, ETEC: Enterotoxigenic *E. coli* susceptibility, FA_C14:0: Myristic acid content, FA_C16:1: Palmitoleic acid content, FA_C18:0: Stearic acid content, FA_C18:1: Oleic acid content, FA_C20:0: Arachidic acid content, FA_C20:1: Cis_11_eicosenoic acid content, FAASR: Arachidic acid to stearic acid ratio, FAPALR: Palmitoleic acid to palmitic acid ratio, FAPMR: Palmitic acid to myristic acid ratio, FEEDIN: Daily feed intake, HCWT: Carcass weight (hot), IMFAT: Intermuscular fat content, LDL: Ldl cholesterol, LEANP: Lean meat percentage, MUFA: Monounsaturated fatty acid content, NUMMF: Number of muscle fibers per unit area, PH: Ph 24 hr post_mortem (loin), PMORT: Piglet mortality, PRRSVL: Prrs viral load, PRRSVS: Prrsv susceptibility, SFA: Saturated fatty acid content, SHEAR: Shear force, SKINT: Skin thickness, TIBL: Tibia length, TNUM: Teat number, TVNUM: Thoracic vertebra number, WT: Body weight (21 weeks).

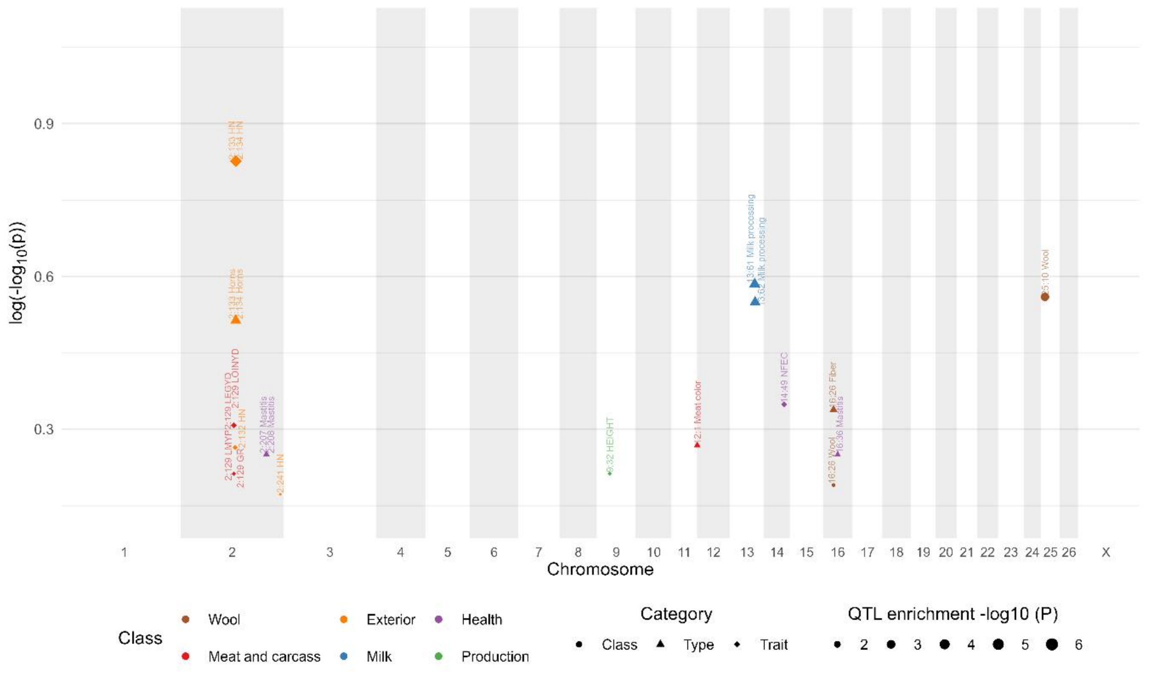

Figure S8. Manhattan plot of QTL enrichment analysis for sheep phenotypic categories. Only significant genomic windows ($P < 0.05$) are displayed. The graph displays the genes located in the most significant genomic regions of each chromosome. For the purposes of graph reduction, the Y-axis represents the logarithm of the negative logarithm (base 10) of the P-values. The traits and their abbreviations are as follows: GR: Soft tissue depth at gr site, HEIGHT: Body height, HN: Horn number, LEGYD: Leg yield, LMYP: Lean meat yield percentage, LOINYD: Loin yield, NFEC: *Nematodirus* fec.

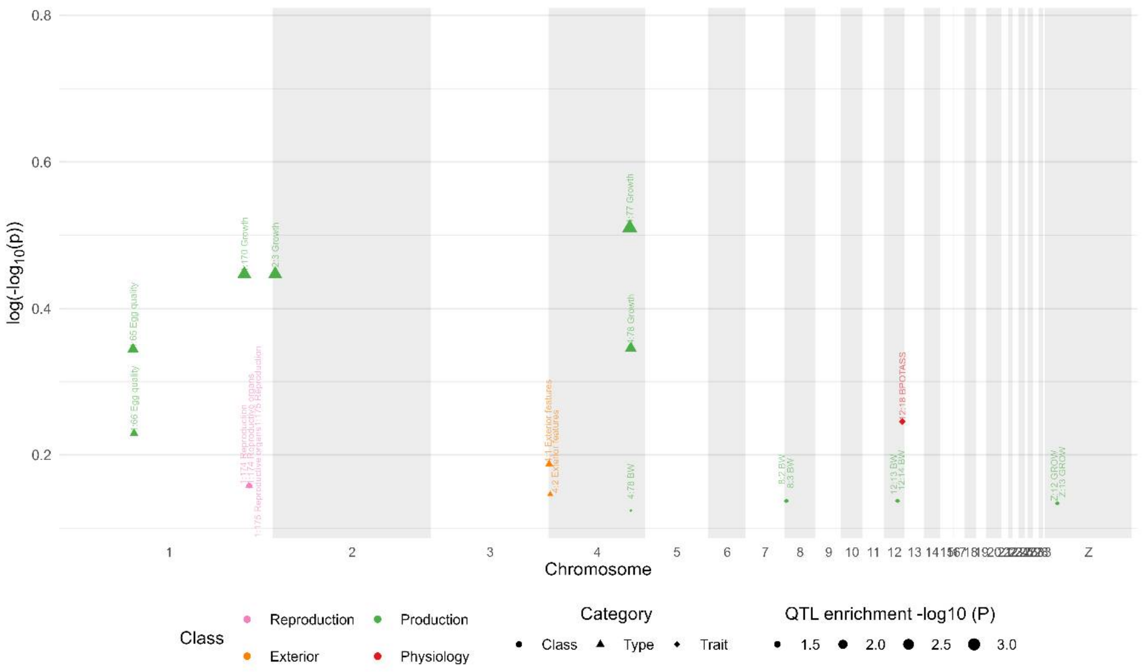

Figure S9. Manhattan plot of QTL enrichment analysis for chicken phenotypic categories. Only significant genomic windows ($P < 0.05$) are displayed. The graph displays the genes located in the most significant genomic regions of each chromosome. For the purposes of graph reduction, the Y-axis represents the logarithm of the negative logarithm (base 10) of the P-values. The traits and their abbreviations are as follows: BPOTASS: Blood potassium level, BW: Body weight (36 days), BW: Body weight (70 days), GROW: Growth (56_84 days), GROW: Growth (84_112 days).

Table S1. Mendelian gene list used for the enrichment analysis.

Table S2. Candidate gene list used for the enrichment analysis.

Table S3. Pearson correlation values between QTL and gene counts across chromosomes and window sizes (0.25, 1, 2.5, and 5 Mbp).

Table S4. Pearson correlation values between QTL and SNP counts across chromosomes and window sizes (0.25, 1, 2.5, and 5 Mbp).

Table S5. Pearson correlation values between genes and SNP counts across chromosomes and window sizes (0.25, 1, 2.5, and 5 Mbp).

Table S6. Observed versus expected QTL by chro-mosome for each genome.

Table S7. Genome-wide enrichment results across the four studied genomes using gene density as the success probability in a binomial test.

Table S8. Genome-wide enrichment results across the four studied genomes using hypergeometric tests for trait-specific analysis
